# MobileNet-WDD: a lightweight deep learning image classification method for identifying defects in wheat grains

**DOI:** 10.3389/fpls.2026.1882943

**Published:** 2026-06-30

**Authors:** Yunzhao Ma, Wenyue Wang, Wenfu Wu, Yan Xu

**Affiliations:** 1 College of Biological and Agricultural Engineering, Jilin University, Changchun, China; 2 School of Grain Engineering and Nutritional Science, Jilin Business and Technology College, Changchun, China; 3 Institute of XinJiang Uygur Autonomous Region Grain and Oil Science (Grain and Oil Product Quality Supervision and Inspection Station of Xinjiang Uygur Autonomous Region), Urumqi, China; 4 College of Food and Strategic Reserves, Henan University of Technology, Zhengzhou, China

**Keywords:** deep learning, image classification, MobileNet, quality testing, wheat grains

## Abstract

To address the challenge of simultaneously optimizing model accuracy and computational efficiency in the automated inspection of wheat grain appearance quality, this study proposes a lightweight deep learning model called MobileNet-WDD. Based on the MobileNetV4-small architecture, this model incorporates the SimAM attention mechanism to enhance feature discrimination capabilities and employs Ghost convolutions and the Mish activation function to optimize the network structure, thereby significantly reducing model complexity while maintaining high recognition accuracy. Using six typical categories of wheat grains—diseased spots, insect damage, mold, sprouting, damage, and intact grains—as the research subjects, experimental results show that compared to the baseline model, MobileNet-WDD achieves a 6.1% increase in accuracy, reaching 94.2%; a 30.3% reduction in the number of parameters; computational cost by 27.6%; and inference speed from 155 FPS to 183 FPS, representing a 1.18-fold acceleration. Quantitative analysis confirms that this model achieves high-precision recognition while offering significant advantages in terms of lightweight design and efficient computational performance, providing an efficient and feasible technical solution for real-time non-destructive inspection of wheat grains.

## 1 Introduction

As one of the world’s most important food crops, the quality of wheat grains directly impacts food security, processing quality, and economic value (Augustin et al., 2016). During harvesting, storage, and processing, wheat grains are susceptible to various biotic and abiotic factors, leading to defects such as disease spots, insect damage, fungal infection, sprouting, and mechanical damage (Singh and Joshi, 2025). These defective grains not only reduce flour yield and quality but may also produce mycotoxins, posing a threat to human health (Neme and Mohammed, 2017; Chen et al., 2023; Stoev, 2024; Godana et al., 2023). Therefore, rapid and accurate multi-category defect identification of wheat grains during grain procurement, grading, and pre-processing is of significant practical importance.

Traditional wheat grain quality inspection primarily relies on manual visual sorting, a method that is not only inefficient and highly subjective but also struggles to ensure consistency and accuracy in large-scale operations. In recent years, machine vision technology—combining traditional image processing with shallow machine learning—has found some application in grain classification (Chen et al., 2025; Yu et al., 2025; Song et al., 2025). However, such methods typically rely on manually designed color, texture, and morphological features, making it difficult to fully characterize complex features or subtle differences between similar defects (Hashmi et al., 2023). They perform particularly poorly when distinguishing easily confused categories such as mold, disease spots, and insect damage. At the same time, some researchers have attempted to use spectroscopic techniques to analyze crop composition and predict crop quality (Zhang et al., 2025; Fomina et al., 2025; Ziegler et al., 2025; Zohrabi et al., 2024; Rózylo et al., 2023; Dewan and Khatkar, 2023; Unuvar et al., 2021; Bec et al., 2020; Pandiselvam et al., 2023; Farber and Kurouski, 2022; Buitrago et al., 2018). While this method offers advantages such as non-destructive testing and rapid analysis, it still faces certain challenges in the rapid, non-destructive identification of large numbers of samples. These challenges include the susceptibility of spectral data to environmental noise, insufficient model generalization across different varieties, and the difficulty of existing equipment in balancing high throughput with high precision (Hernanda et al., 2023). These factors limit the direct application of spectroscopic techniques in large-scale, real-time crop identification scenarios (García-Vera et al., 2024).

However, existing deep learning-based grain detection models generally face a trade-off between accuracy and efficiency (Huang et al., 2025; Meyering et al., 2025). On the one hand, in pursuit of high recognition accuracy, researchers often employ deep neural networks with complex structures and a large number of parameters, resulting in high computational costs and slow inference speeds, making it difficult to deploy these models on embedded devices or real-time production lines with limited computational resources (Yu et al., 2024; Wang et al., 2026; Lamba et al., 2023). On the other hand, while lightweight models can achieve fast inference, they often suffer from reduced accuracy in fine-grained classification tasks due to insufficient feature representation capabilities (Li et al., 2024; Arya and Mishra, 2024; Sun et al., 2025; Wang et al., 2025; Guan et al., 2023). This is particularly true for wheat grain categories with similar appearances or subtle defects, where recognition performance is often unsatisfactory (Farmonov et al., 2024). Therefore, how to achieve model lightweighting and efficient inference while ensuring high accuracy has become a critical issue that urgently needs to be addressed in the field of automated wheat grain detection.

As shown in **Table 1**, numerous studies have been conducted on crop classification tasks in recent years. Existing research covers various major crops such as rice, maize, soybean, and wheat. Adopting diverse algorithms including convolutional neural networks, Transformer, and traditional machine learning methods, these approaches achieve excellent classification accuracy of over 90% on various crop datasets, with the best-performing models reaching up to 99.1% accuracy. These results fully validate the feasibility and effectiveness of deep learning and intelligent algorithms for crop classification and recognition, laying a solid foundation for intelligent crop identification and agricultural remote sensing monitoring. Nevertheless, in-depth analysis indicates that current state-of-the-art crop classification models still present obvious practical limitations. Most existing high-accuracy models suffer from complex network structures and excessive parameters. Although they achieve promising classification performance, they exhibit low inference efficiency and slow computational speed, while imposing high requirements on hardware computing resources. Such models are difficult to adapt to low-computing and real-time agricultural scenarios based on field mobile and edge devices, and fail to meet the large-scale, real-time, and lightweight requirements of modern agricultural applications, which greatly restricts the practical deployment and large-scale promotion of crop classification algorithms.

**Table 1:** Status of crop classification in recent years.

No.	Crop	Accuracy	Data volume	Class	Method	References
1	Soybean	97.84%	7052	6	MobileNetV2	(Zhao et al., 2021)
2	Rice	100%	75000	5	CNN	(Koklu et al., 2021)
3	Rice	89.4%	1680	8	PointNet	(Qian et al., 2021)
4	Soybean	96.2%	8011	5	SoybeanNet	(Huang et al., 2022)
5	Wheat	97.6%	21000	7	QSVM	(Fazel-Niari et al., 2022)
6	Maize	96.47%	32500	19	Transformer	(Bi et al., 2022)
7	Wheat	97%	1750	7	BP	(Zhu et al., 2023)
8	Maize	96.65%	735	7	CNN	(Zhang et al., 2023)
9	Tomato	96.09%	3200	4	BiLSTM	(Sabanci, 2023)
10	Maize	91.23%	5877	6	ResNet50	(Li et al., 2024)
11	Maize	98.36%	129230	8	ERNet	(Li et al., 2024)
12	Maize	95.9%	1650	11	RF&GWO	(Bi et al., 2024)
13	Rice	95.13%	30000	10	ResNet50	(Yu et al., 2024)
14	Rice	97.12%	59840	6	IDKNN	(Ge et al., 2024)
15	Maize	99.1%	10000	20	CNN	(Zhang et al., 2024)
16	Rice	92.00%	36735	19	FasterNet	(Yu et al., 2025)
17	Mung Bean	94.01%	34890	8	MobileNetV2	(Song et al., 2025)
18	Rice	98.81%	12000	9	ResNeXt50	(Chen et al., 2025)
19	Garlic	98.95%	1461	3	ResNet34	(He et al., 2026)

To address the aforementioned critical challenges of excessive parameters, heavy computational burden, and low real-time inference efficiency inherent in existing high-precision crop classification models, this study proposes a novel lightweight end-to-end deep learning model termed MobileNet-WDD for fine-grained wheat grain quality classification. Unlike conventional complex models that pursue accuracy at the cost of computational efficiency, the proposed model adopts MobileNetV4-small (Qin et al., 2024) as the efficient backbone to retain fundamental feature extraction capability with compact architecture. To compensate for the feature degradation caused by lightweight design and enhance fine-grained classification performance, a parameter-free SimAM attention mechanism (Yang et al., 2021) is embedded to strengthen feature discrimination, enabling the network to precisely focus on the subtle and discriminative visual regions of wheat grains. Furthermore, the standard point-wise convolution is replaced with Ghost convolution (Han et al., 2020) to generate sufficient redundant feature maps at extremely low computational cost, which effectively reduces overall model parameters and computational complexity while preserving comprehensive feature information. Additionally, the non-monotonic Mish activation function (Misra, 2019) is adopted to replace the traditional ReLU function, optimizing gradient propagation and improving the nonlinear feature representation ability of the lightweight network. Based on a self-constructed six-category wheat grain image dataset, rigorous ablation experiments, comparative analyses of different attention mechanisms, activation functions, and state-of-the-art (SOTA) model comparisons are conducted to fully verify the effectiveness and superiority of each designed module and the overall model performance. The experimental results demonstrate that the proposed MobileNet-WDD achieves a competitive classification accuracy of 94.2%, with only 2.65 million parameters and a low computational cost of 0.42 GFLOPs, while reaching a high inference speed of 183 FPS. Compared with baseline and existing SOTA models, our method realizes an optimal trade-off between classification accuracy and computational efficiency, perfectly solving the contradiction between high recognition performance and lightweight deployment requirements in agricultural crop classification tasks. The core contributions of this study are summarized as follows:

1. A novel lightweight MobileNet-WDD model is proposed for fine-grained wheat grain appearance quality classification, which achieves superior comprehensive performance on the six-class wheat grain classification task and effectively overcomes the low inference efficiency and heavy computing defects of traditional crop classification models.
2. The synergistic enhancement mechanism of SimAM attention, Ghost convolution, and Mish activation function is systematically explored and analyzed for fine-grained agricultural classification tasks, providing a feasible lightweight optimization paradigm and valuable reference for the structural design of similar agricultural intelligent detection models.
3. Multi-dimensional comparative experiments fully validate that MobileNet-WDD achieves excellent balanced performance in classification accuracy, parameter scale, computational complexity, and inference speed, possessing great practical application value and deployment potential for real-time and low-cost agricultural field detection systems.

The remainder of this paper is organized as follows. Section 2 presents the materials and methods adopted in this study, which elaborates the detailed information of the constructed wheat grain dataset and the specific structural design, module composition and optimization principles of the proposed MobileNet-WDD model. Section 3 illustrates the experimental results and corresponding analyses, comprehensively evaluating the overall performance of the proposed model on the wheat grain dataset through sufficient ablation experiments and multi-group comparative experiments with state-of-the-art methods. Section 4 discusses the limitations of the current study and puts forward reasonable and targeted future research directions for further optimization and practical application of agricultural lightweight classification models. Finally, Section 5 concludes the entire research work and summarizes the core findings and application value of this study.

## 2 Materials and methods

### 2.1 Data acquisition and preprocessing

**Figure 1:**
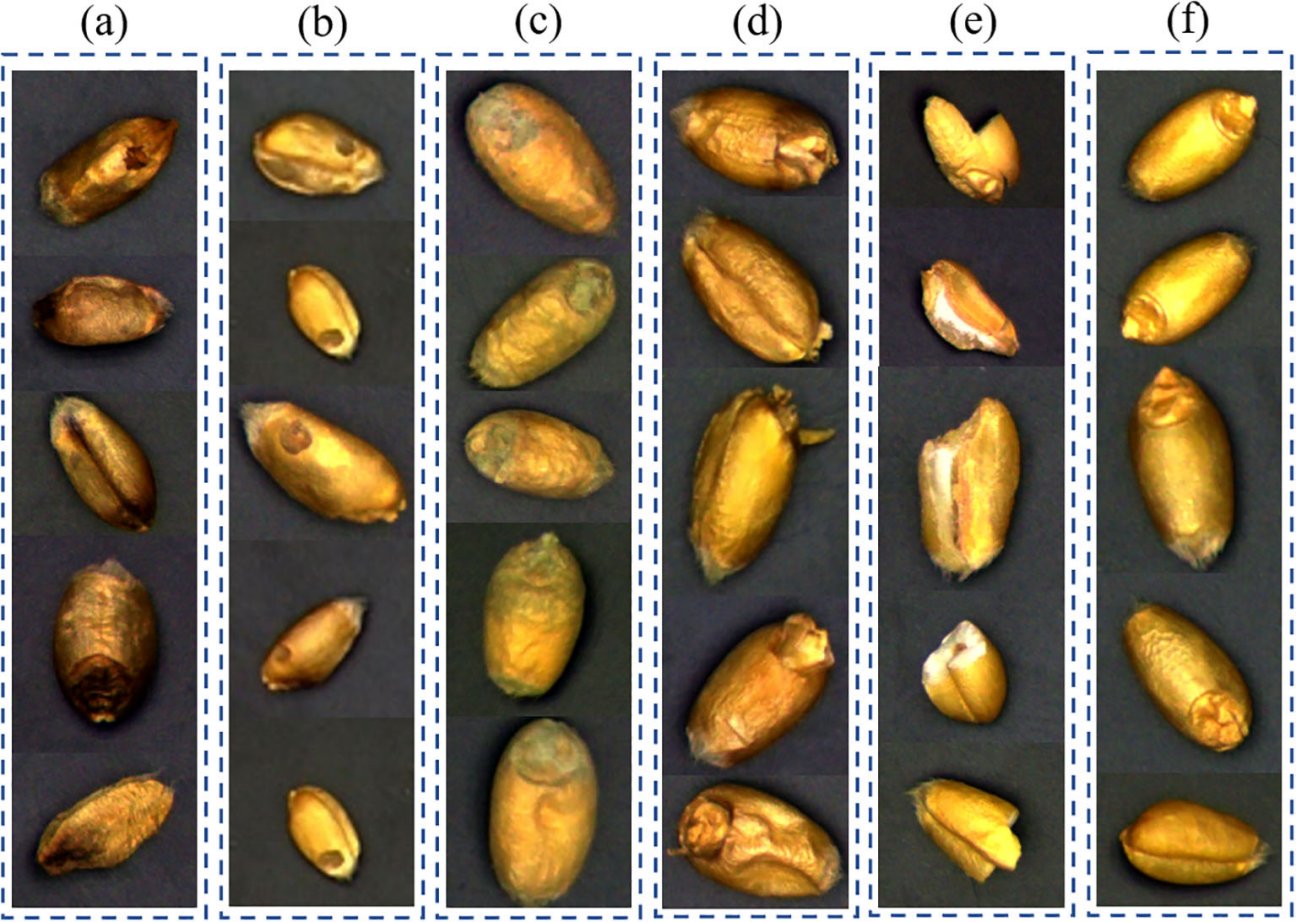
Wheat grain dataset: **(a)** diseased spots, **(b)** insect damage, **(c)** mold, **(d)** germination, **(e)** damage, **(f)** intact. During model training, **(a-f)** were labeled as 1 through 6, respectively.

**Figure 1** shows six typical types of wheat samples from the wheat grain dataset. Among them, diseased grains exhibit discolored or necrotic patches on their surfaces caused by fungal or bacterial infections; insect-damaged grains have holes or defects resulting from feeding by storage pests; moldy grains are covered with visible mycelium or spores and display abnormal coloring; germinated grains have prematurely sprouted due to excessive humidity or improper storage, with the embryo noticeably elongated; damaged grains have cracked seed coats or exposed endosperm resulting from mechanical threshing or processing; intact grains, on the other hand, have smooth surfaces, normal coloration, and no visible defects.

The fundamental reason for the detailed classification of these categories lies in the fact that wheat, as a major global staple food and industrial raw material, has grain quality that directly impacts flour yield, nutritional safety, and processing characteristics. Grains with lesions, insect damage, or mold may carry mycotoxins, posing risks to human and livestock health; increased amylase activity in sprouted grains can cause baked goods to become sticky and degrade in texture; damaged grains are prone to moisture absorption and mold growth during storage, and they affect the ash content and color of the milled flour. By using computer vision models to automatically identify and sort these categories, rapid, non-destructive wheat quality inspection can be achieved, reducing human subjective errors, ensuring quality consistency throughout grain storage, processing, and trade, and providing a reliable basis for subsequent targeted treatment.

To ensure consistency and controllability in image acquisition, the entire imaging process was conducted in a darkroom environment. A black light-absorbing cloth was laid as the background to eliminate stray light reflections and highlight the grain’s outline. The Honor Magic6 smartphone was selected as the imaging device, mounted on a top bracket, and positioned vertically to capture images of the samples, with the lens set at a vertical distance of 20 centimeters from the grain surface. To enhance the diversity of the dataset and the model’s generalization ability in real-world scenarios, this study further integrated publicly available wheat grain image datasets with the self-built dataset. The dataset contains 2000 images for each category, consisting of self-collected and public images at a 1:1 ratio. We firstly divided all data into training, validation and test sets following a 6:2:2 split. Online data augmentation was then exclusively applied to the training set, including random angle rotation, Gaussian noise injection and random brightness adjustment, to mimic complex field imaging environments. These sets were finally used for model training, fine-tuning and performance evaluation respectively.

### 2.2 Model building

**Figure 2:**
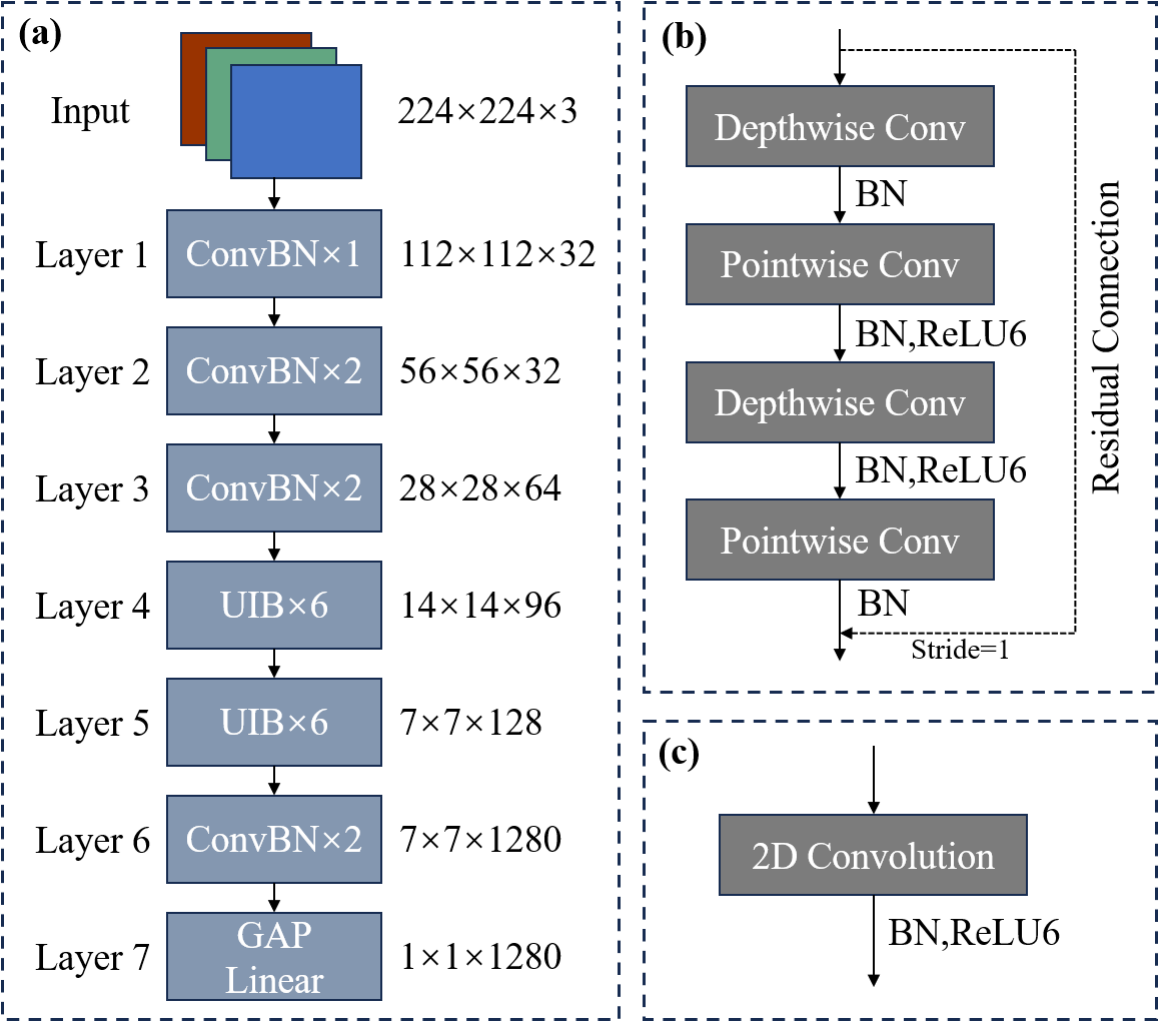
Schematic diagram of the overall architecture of MobileNetV4-small: **(a)** the backbone architecture of MobileNetV4-small; **(b)** the UIB architecture; **(c)** the ConvBN architecture.

This study focuses on the rapid identification of anomalous wheat grains and adopts the lightweight convolutional neural network MobileNetV4−small as the backbone for feature extraction. Proposed by Google in 2024, MobileNetV4 represents a new generation of mobile-oriented models that achieve high inference efficiency while being systematically optimized for diverse hardware platforms. Compared to earlier lightweight models used for agricultural image classification, the MobileNetV4 architecture features a more advanced and flexible design. By leveraging a novel universal inverted bottleneck module, it enhances feature extraction capabilities. Combined with a lightweight attention mechanism, it can better capture subtle and blurry crop defect features in field images. Additionally, the model has been specifically optimized for various edge hardware devices, resulting in more stable inference performance. While maintaining its lightweight advantages, it effectively addresses the issue of insufficient accuracy in traditional lightweight models when applied to complex agricultural scenarios, making it better suited for deployment on agricultural edge devices in the field. Among its variants, MobileNetV4−small has the smallest parameter count, making it particularly suitable for deployment on portable field devices or embedded platforms to meet real−time detection requirements. As illustrated in **Figure 2a**, the network takes as input an RGB image of resolution 224×224×3. Layers 1 to 3 employ a standard ConvBN block for initial downsampling and feature extraction. Layers 4 and 5 stack Universal Inverted Bottleneck (UIB) modules to enhance feature representation under lightweight constraints. Layer 6 adopts a high−dimensional ConvBN block for further feature fusion. Finally, the classification result is produced via global average pooling (GAP) followed by a fully connected (Linear) layer. The dimensions and channel numbers of the feature maps at each layer are annotated on the right.

As shown in **Figure 2b**, UIB is a lightweight convolutional unit developed from the conventional inverted bottleneck structure. Its core architecture is built upon depthwise separable convolutions. Within the inverted bottleneck framework formed by 1×1 pointwise convolutions (PW), configurable front and middle depthwise convolutions (DW) are introduced, resulting in a flexible and general-purpose structure. Specifically, the input features may first pass through an optional front depthwise convolution for spatial preprocessing, followed by a 1×1 pointwise convolution for channel expansion. In the resulting high-dimensional feature space, an optional middle depthwise convolution is then applied to extract spatial features. Finally, another 1×1 pointwise convolution performs channel compression and projection. When the input and output channel dimensions match, a residual connection is added to enable cross-layer feature reuse. Each convolutional layer is accompanied by batch normalization (BN) and the ReLU6 activation function for feature normalization and nonlinear transformation. This design preserves the advantages of depthwise separable convolutions—lightweight and low computational cost—while enhancing spatial feature extraction capability through the configurable dual-depthwise convolution design without significantly increasing the number of parameters. Moreover, it can adapt to the inference requirements of different hardware platforms via neural architecture search, serving as a core module for mid- to high-level feature extraction in lightweight convolutional neural networks.

Therefore, this study employs MobileNetV4-small as the base model for wheat grain anomaly detection. This model utilizes a lightweight UIB as its core architecture, which, while maintaining a minimal number of parameters and computational load, possesses excellent fine-grained feature extraction capabilities. It can accurately capture subtle abnormalities in the appearance, texture, and color of wheat grains, effectively distinguishing normal grains from abnormal types such as moldy, damaged, insect-eaten, and diseased grains. MobileNetV4-small features fast inference speeds and low hardware dependency, enabling efficient operation on mobile devices and edge devices. It is particularly well-suited for rapid detection and on-site deployment in practical scenarios such as fields and grain warehouses. While ensuring the accuracy of abnormal grain recognition, it significantly enhances detection efficiency and practicality, providing a lightweight, efficient, and stable foundational model for rapid screening and intelligent grading of wheat quality.

**Figure 3:**
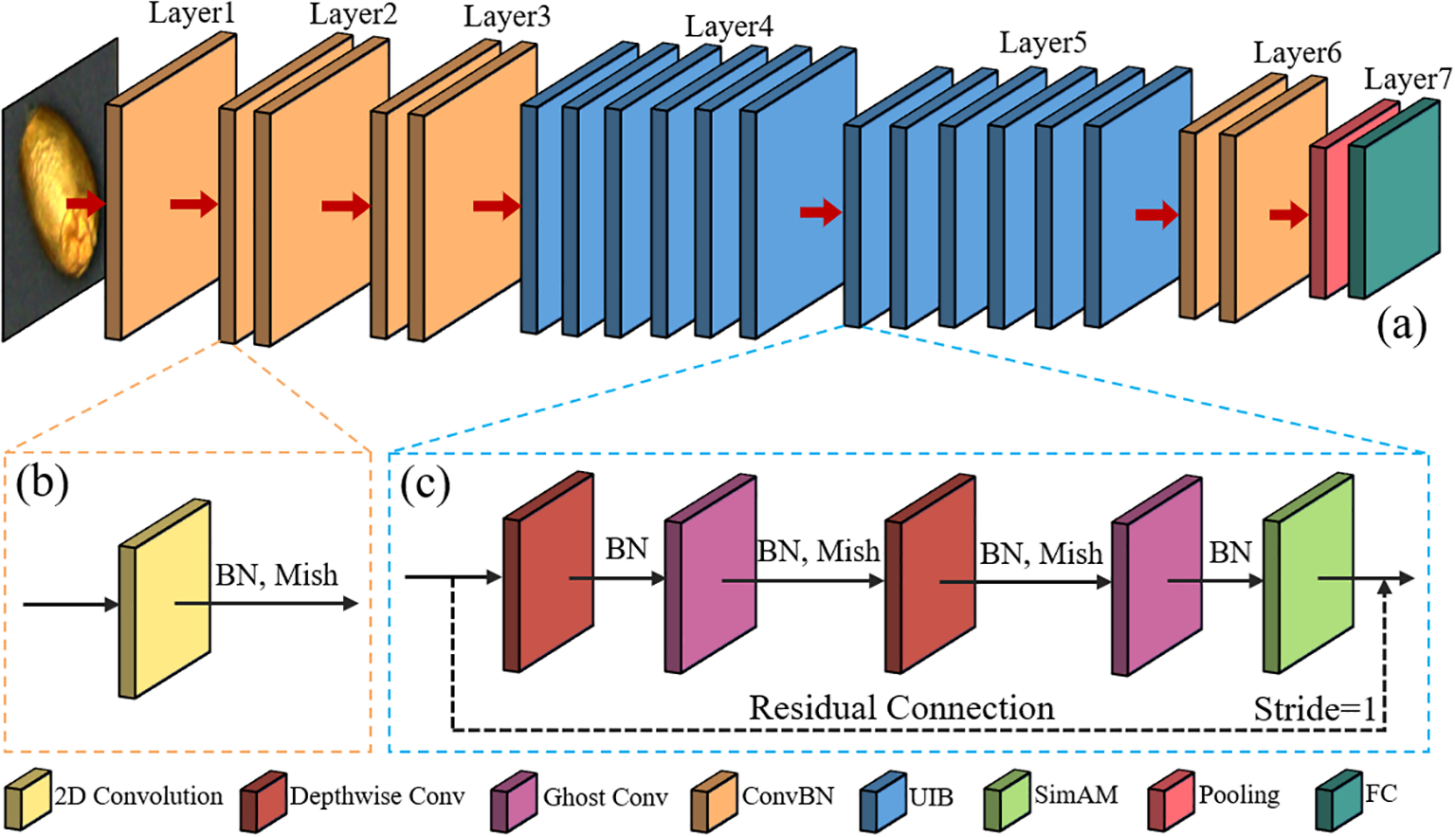
Schematic diagram of the overall architecture of MobileNet-WDD: **(a)** the backbone architecture of MobileNet-WDD; **(b)** the improved ConvBN architecture; **(c)** the improved UIB architecture.

To further improve the accuracy and efficiency of wheat grain anomaly detection, this study uses MobileNetV4-small as the base network and performs multi-dimensional optimizations and improvements to the model architecture, taking into account the detection requirements for wheat grain anomalies, which feature minute details and complex categories. The improved model architecture is shown in **Figure 3**. In the core UIB unit, the original point-wise convolution is replaced with Ghost convolution. By generating both base features and low-cost Ghost features, the model significantly reduces redundant computations and the number of parameters while retaining key feature information, making it more suitable for edge and mobile deployment while maintaining its lightweight characteristics. Additionally, a SimAM attention mechanism was introduced after each UIB module. This mechanism enables adaptive focusing on important feature regions without requiring additional parameters, effectively enhancing the model’s ability to extract features from subtle abnormal areas on wheat grain surfaces—such as lesions, damage, mold, and insect damage—and improving the model’s classification accuracy for abnormal targets in complex backgrounds. Furthermore, the ReLU6 activation function in the network is replaced with the Mish activation function. By leveraging the Mish activation function’s advantages of smoothing non-monotonicities and providing more stable information propagation, the model’s gradient propagation during training is enhanced, mitigating the vanishing gradient problem in deep networks, and improving the model’s fitting capability and generalization performance in fine-grained classification tasks. Following these improvements, the model retains its lightweight and efficient nature while possessing stronger feature representation and anomaly detection capabilities, thereby better meeting the practical application requirements for rapid and accurate detection of wheat grain anomalies.

**Figure 4:**
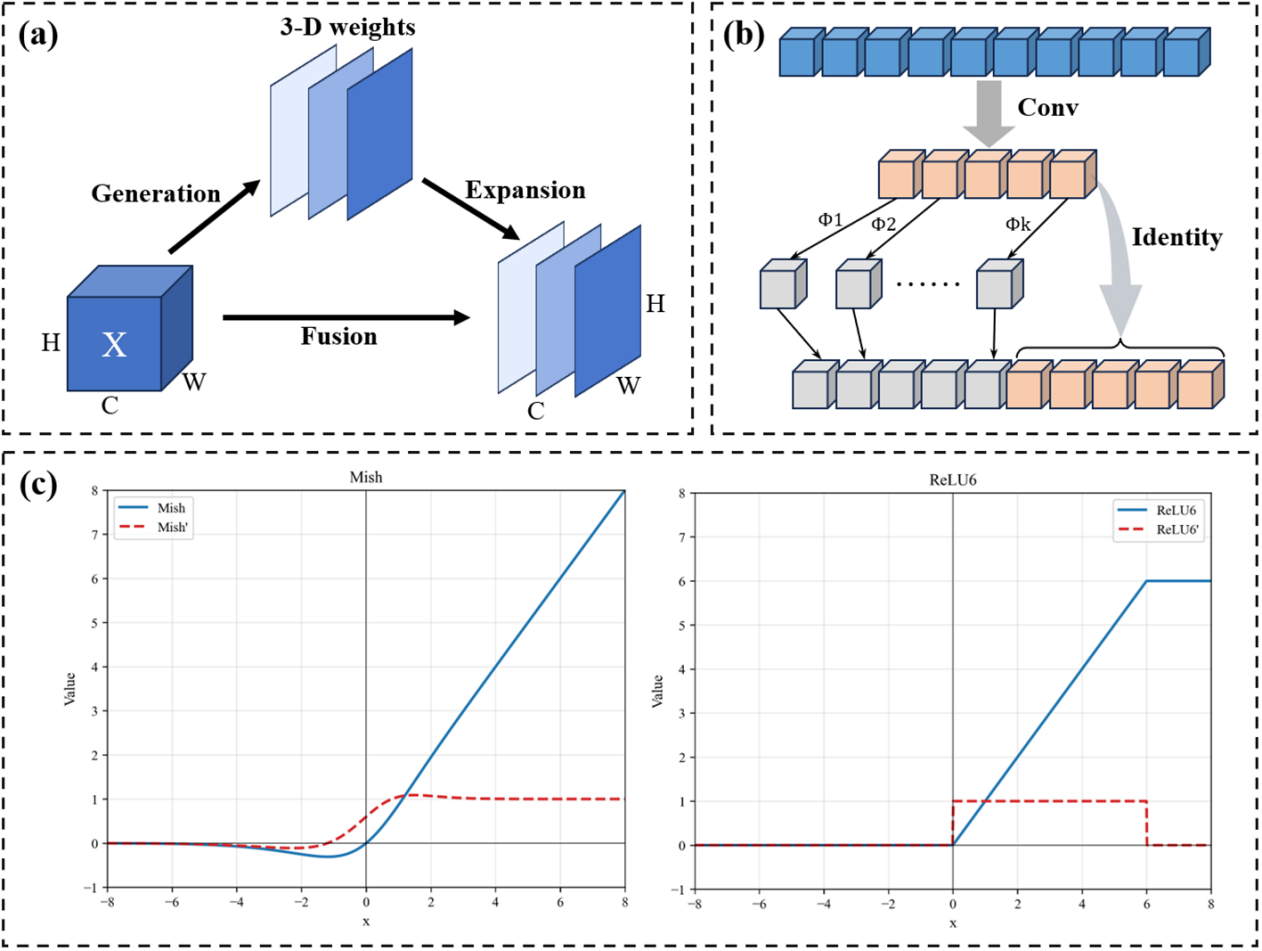
Schematic diagram of the improved architecture in MobileNet-WDD: **(a)** Schematic diagram of the SimAM architecture; **(b)** Schematic diagram of the Ghost Conv architecture; **(c)** Curves of the Mish and ReLU6 functions, along with their derivative curves.

**Figure 4a** shows a schematic diagram of the SimAM (Simple, Parameter-Free Attention Module), a parameter-free, lightweight, and efficient attention mechanism. Its core principle is based on the neuroscientific concept that “information-rich neurons exhibit significant differences from their neighboring neurons.” By quantifying the importance of each neuron through an energy function, it achieves adaptive focusing on key regions of feature maps without increasing the number of model parameters or computational load. Its core energy function is shown in Equation 1.

(1) $$e_{t}=\frac{4\left(\sigma^{2}+\lambda \right)}{{\left(t-\mu_{t}\right)}^{2}+2\sigma_{t}^{2}+2\lambda }$$

Here, t represents the current neuron’s feature value; *μ_t_* and $\sigma_{t}^{2}$ denote the mean and variance, respectively, of all neurons in the same channel except t; and λ is a small regularization constant used to prevent the denominator from becoming zero and ensure numerical stability. The smaller the energy value *e_t_*, the greater the difference between that neuron and its neighbors, indicating that the information is more important and corresponding to a higher attention weight. The energy value is then normalized to an attention weight using the sigmoid function, as shown in Equation 2.

(2) $$A_{t}=\frac{1}{1+\exp\left(-e_{t}\right)}$$

The final output is obtained by element-wise multiplication of the original features and the attention weights, as shown in Equation 3.

(3) $$\hat{X}=X⊙A$$

Here, A represents the final output attention weights, X represents the original feature map, and ⊙ denotes element-wise multiplication. This mechanism requires no additional learnable parameters and can be directly embedded into each layer of a convolutional network, simultaneously enhancing key features in both the channel and spatial dimensions to meet the requirements for precise identification of subtle anomalies in wheat grains.

**Figure 4b** illustrates the structural diagram of Ghost Conv. The core idea of Ghost Conv is to generate the original feature map using a small number of standard convolutions, and then utilize low-cost linear operations to generate additional redundant ghost features, thereby reducing computational complexity while maintaining expressive power. Let the input feature be $X\in {\mathbb{R}}^{C\times H\times W}$. First, a standard convolution with m channels is applied to obtain the base feature map $Y^{\prime }\in {\mathbb{R}}^{m\times H\times W}$, as shown in Equation 4.

(4) $$Y^{\prime }=X*f_{1}$$

Here, *f*_1_ represents the convolution kernel. Subsequently, a channel-wise linear transformation is applied to each channel of Y′ to generate s−1 phantom features, which are then concatenated to produce the complete output feature $Y\in {\mathbb{R}}^{n\times H\times W}$, where n = m×s. The overall process can be expressed as Equation 5.

(5) $$Y=Concat\left(Y^{\prime },{\text{Φ}}_{1}\left(Y_{1}^{\prime }\right),{\text{Φ}}_{2}\left(Y_{2}^{\prime }\right),\ldots \ldots ,{\text{Φ}}_{m\left(s-1\right)}\left(Y_{m}^{\prime }\right)\right)$$

Here, Φ*_i_* represents a parameter-free or low-cost linear operation that does not require a large number of additional convolution kernel parameters, thereby enabling efficient feature extraction from wheat grains.

In the design of activation functions for deep learning, ReLU6 and Mish represent two distinct approaches. The mathematical expression for ReLU6 is shown in Equation 6.

(6) ReLU6(x)=min(max(0,x),6)

The function curve consists of three segments: it is constant at 0 when 0 ≤ x ≤ 6, increases linearly (with a slope of 1) when 0< x< 6, and is truncated to the constant 6 when x ≥ 6. The corresponding derivative curve is a piecewise constant, as shown in Equation 7.

(7) $$\frac{d}{dx}\text{ReLU}6\left(x\right)=\{\begin{array}{cc} 0, & x<0,\\ 1, & 0<x<6,\\ 0, & x>6, \end{array}$$

Furthermore, it is not differentiable at x = 0 and x = 6. In contrast, Mish’s mathematical expression is given by Equation 8.

(8) Mish(x)=x·tanh(softplus(x))

The expression for the *softplus*(x) function is shown in Equation 9, while tanh(x) is the hyperbolic tangent function, whose mathematical expression is shown in Equation 10.

(9) $$softplus\left(x\right)=ln\left(1+e^{x}\right)$$

(10) $$\tanh\left(x\right)=\frac{e^{x}-e^{-x}}{e^{x}+e^{-x}}$$

The graph of the Mish function is smooth and non-monotonic; it lies slightly below zero on the negative half-axis (with a minimum of approximately -0.31 at x≈-1.2), then rises smoothly through the origin, gradually approaching a linear trend on the positive half-axis while remaining slightly below y=x, and has no upper bound. The expression of its derivative curve is as shown in Equation 11, and it is also smooth and continuous.

(11) $$\frac{d}{dx}Mish\left(x\right)=\tanh\left(softplus\left(x\right)\right)+x\cdot sech^{2}\left(softplus\left(x\right)\right)\cdot \sigma \left(x\right)$$

Here, σ(*x*) denotes the sigmoid function, whose mathematical expression is shown in Equation 12.

(12) $$\sigma \left(x\right)=\frac{1}{\left(1+e^{-x}\right)}$$

**Figure 4c** shows the curves of the two activation functions and their derivative curves. A comparison of the curves clearly demonstrates the advantages of Mish: the derivative of ReLU6 is constant (1 or 0) in the non-zero region, lacking gradient information for negative inputs, and the hard clipping at x=6 limits the range of feature expression; In contrast, Mish is differentiable everywhere across the entire real number domain. Its slightly negative outputs in the negative region preserve a faint gradient flow, which helps mitigate neuron death, while its non-monotonicity allows for better information flow and richer feature mappings. Furthermore, Mish’s smoothness makes the optimization process more stable, particularly in deep networks; it typically yields higher accuracy and faster convergence rates than ReLU6.

### 2.3 Training strategies

#### 2.3.1 Cross-entropy loss function

This study employs the cross-entropy loss function to quantify the discrepancy between the model’s predictions and the true labels. This function is a standard tool for measuring model performance in classification tasks; its theoretical foundation stems from information theory and is used to measure the divergence between two probability distributions. Specifically, let P denote the distribution of the true labels and Q denote the probability distribution predicted by the model. For a single sample, the mathematical expression for the cross-entropy loss L is shown in Equation 13.

(13) $$ℒ\left(P,Q\right)=-\sum_{i}P\left(i\right)\log Q\left(i\right)$$

In practical multi-class classification tasks, since real-world labels typically use one-hot encoding—where the probability P(c) is 1 for the correct class and 0 for all other classes—the above formula can be simplified to a calculation of the log-probability for the correct class only, as shown in Equation 14.

(14) ℒ=−log(Q(c))

Here, Q(c) represents the probability that the model predicts the sample belongs to the true class c. By calculating this loss, we can evaluate the model’s confidence in its predictions for each sample; the smaller the loss value, the more accurate the model’s prediction of the true class.

#### 2.3.2 Cosine annealing algorithm

This study employs the Cosine Annealing algorithm to dynamically adjust the learning rate during model training. Inspired by the physical annealing process, the Cosine Annealing algorithm simulates the behavior of molecules in a solid at high temperatures—where they move freely and gradually stabilize as the temperature decreases—to dynamically adjust the learning rate during the optimization process. The core idea of this algorithm is as follows: a larger learning rate is used in the early stages of training to enable the model to rapidly explore the solution space and effectively overcome local minima and saddle points; as training progresses, the learning rate gradually decreases according to the pattern of a cosine function, allowing the model to perform fine-tuning as it approaches the optimal solution, thereby improving training stability and enhancing the model’s generalization performance. The cosine annealing strategy dynamically calculates the learning rate *η_t_* corresponding to the current iteration step *T_cur_* using Equation 15.

(15) $$\eta_{t}=\eta_{min}+\frac{1}{2}\left(\eta_{max}-\eta_{min}\right)\left(1+\cos\left(\frac{T_{cur}}{T_{max}}\pi \right)\right)$$

Here, *η_t_* is the learning rate at the current time step; *η_min_* and *η_max_* represent the lower and upper bounds of the learning rate, respectively; *T_cur_* is the current iteration count; and *T_max_* is the total number of iterations within a single cycle, which corresponds to half a cycle of the cosine curve. This formula causes the learning rate to start from the initial value *η_max_* and smoothly decrease to *η_min_* following the shape of a cosine curve. Additionally, this study introduces a restart strategy into the cosine annealing algorithm: after completing a full cycle, the learning rate returns to a higher value or oscillates near the minimum value, thereby helping the model escape local optima and find a better global solution.

By incorporating a cosine-smoothed annealing strategy, the model in this study is able to converge rapidly during the early stages of training using a higher learning rate, and then fine-tune its parameters in the later stages by gradually reducing the learning rate. This dynamic adjustment mechanism effectively avoids the issues of oscillation caused by excessively high learning rates or slow convergence caused by excessively low learning rates in traditional gradient descent methods, thereby achieving superior model performance with limited training resources.

### 2.4 Evaluation criteria

In supervised learning, confusion matrices serve as a fundamental tool for evaluating classification model performance. The matrix organizes predictions against ground truth labels: columns correspond to predicted classes, while rows represent actual classes. For a binary classification task, the matrix consists of four key components. True Positives (TP): Cases where both the actual and predicted labels are positive, False Positives (FP): Negative instances incorrectly predicted as positive, False Negatives (FN): Positive instances misclassified as negative, True Negatives (TN): Correctly identified negative cases. The structure of a binary confusion matrix is illustrated in **Table 2**.

**Table 2:** Confusion matrix of the binary classification problem.

Confusion matrix		Actual results	
		Positive	Negative
Forecast Results	Positive	TP	FP
	Negative	FN	TN

Accuracy (Acc), Precision (P), Recall (R), and F1-score (F1) are derived from the confusion matrix and serve as key metrics for evaluating the classification performance of a model. The corresponding formulas and brief descriptions of these metrics are provided in **Table 3**.

**Table 3:** Brief introduction to evaluation metrics.

Evaluation metrics	Formula	Brief description
Accuracy (Acc)	$Acc=\;\frac{TP+TN}{TP+FP+FN+TN}$	The ratio of the number of correctly predicted positive and negative samples to the total number of samples.
Precision (P)	$P=\;\frac{TP}{TP+FP}$	The ratio of the number of correctly predicted positive samples to the total number of samples predicted to be positive.
Recall (R)	$R=\;\frac{TP}{TP+FN}$	The ratio of the number of correctly identified positive samples to the total number of actual positive samples.
F1-score (F1)	$F1=\;\frac{2TP}{2TP+FP+FN}$	The harmonic mean of precision and recall.

When evaluating lightweight deep learning models, the following four key metrics are typically considered to assess the model’s optimization direction, computational cost, memory usage, and real-time throughput. Together, these metrics guide optimization efforts for resource-constrained deployment environments.

Loss: Measures the model’s prediction error, reflecting its ability to fit the target task. Lower loss indicates higher accuracy, which must be balanced against efficiency metrics to avoid over-simplification.
Parameter Count (Params): The number of trainable parameters, directly impacting memory usage and computation. Lightweight models reduce this via pruning and architectural design to lower storage and computational overhead.
Floating-Point Operations (FLOPs): Quantifies the computational complexity per forward pass. Reducing FLOPs lowers energy consumption, improving suitability for low-power devices.
Frames Per Second (FPS): Indicates throughput—samples processed per second. Higher FPS enables efficient real-time video stream handling and batch processing.

Together, these four metrics form a core evaluation framework for lightweight models, helping researchers balance accuracy and efficiency to meet the deployment demands of edge computing and mobile AI applications.

### 2.5 Experimental environment and hyperparameter settings

The proposed network was trained with input images resized to 224×224 pixels, a batch size of 32, and a base learning rate of 0.01. The optimizer was stochastic gradient descent (SGD), and training ran for 100 iterations. All experiments used a consistent hardware and software setup: a Windows 10 workstation with an Intel Xeon Gold 6246R CPU (3.4 GHz) and an NVIDIA Quadro RTX 8000 GPU (48 GB VRAM). The software environment included Anaconda3 (2021.11), PyCharm IDE, Python 3.8.3, and PyTorch 1.2.1.

## 3 Results and analysis

### 3.1 Hyperparameter optimization and analysis

To verify the effectiveness of the dynamic learning rate adjustment in the cosine annealing algorithm used in this study, we conducted the following validation using the MobileNetV4-small model on the wheat grain dataset: after training with different learning rate scheduling strategies, we evaluated the performance metrics on the validation set. All results represent the average of three independent runs to ensure the authenticity and validity of the data.

As shown in **Table 4**, the cosine annealing algorithm achieved the best results across all evaluation metrics, with an accuracy of 88.07% and a minimum loss of 0.31. Additionally, the precision, recall, and F1 score reached 87.9%, 88.1%, and 88.0%, respectively. This is attributed to the cosine annealing algorithm’s use of a smooth cosine function to periodically adjust the learning rate throughout the training cycle: a higher learning rate in the early stages accelerates convergence, a gradual decline in the middle stages allows for fine-grained exploration of the loss landscape, and a low learning rate in the final stages prevents overshooting the optimal solution. Compared to fixed learning rates, stepwise decay, exponential decay, polynomial decay, and cyclic learning rates, the continuous and smooth decay strategy of cosine annealing significantly improves the model’s generalization ability and training stability. It is particularly suitable for tasks such as the fine-grained classification of wheat grains, where inter-class differences are minimal, and the loss surface is complex.

**Table 4:** Comparison of dynamic learning rate adjustment strategies.

Learning rate adjustment strategy	Initial learning rate	Acc (%)	Loss	P (%)	R (%)	F1 (%)
Fixed value	0.01	83.5	0.46	83.1	83.5	83.3
Stepwise Regression	0.01	85.8	0.39	85.4	85.8	85.6
Exponential Decay	0.01	86.2	0.37	86.0	86.2	86.1
Polynomial Decay	0.01	86.9	0.35	86.7	86.9	86.8
Cyclic Learning Rate	0.01	86.5	0.36	86.3	86.5	86.4
Cosine Annealing Algorithm	0.01	88.1	0.31	87.9	88.1	88.0

### 3.2 Ablation experiment

To validate the contributions of the SimAM, Ghost Conv, and Mish modules, ablation experiments were conducted using MobileNetV4-small as the baseline architecture. Each module was incrementally integrated to assess its individual and synergistic effects on model performance. The results are summarized in **Table 5**. The baseline model attained a precision of 88.1%, a recall of 87.9%, an F1 score of 88.0%, and an accuracy of 88.1%, with 3.80 million parameters, a computational complexity of 0.58 GFLOPs, and an inference speed of 155 FPS.

**Table 5:** Results of the ablation experiment.

SimAM	Ghost conv	Mish	Acc (%)	P (%)	R (%)	F1 (%)	Params (M)	GFLOPs	FPS
			88.1	87.9	88.1	88.0	3.80	0.58	155
✓			92.4	92.2	92.6	92.4	3.80	0.58	153
	✓		88.7	88.5	88.9	88.7	2.65	0.42	178
		✓	91.5	91.3	91.7	91.5	3.80	0.58	154
✓	✓		92.9	92.7	93.1	92.9	2.65	0.42	176
	✓	✓	92.1	91.9	92.3	92.1	2.65	0.42	174
✓		✓	93.0	92.8	93.2	93.0	3.80	0.58	153
✓	✓	✓	94.2	94.3	94.2	94.2	2.65	0.42	183

With the exclusive incorporation of SimAM, the accuracy rose to 92.4%, representing a 4.3 percentage point gain over the baseline. Precision, Recall, and F1 score exhibited improvements of approximately 4% each. Notably, the parameter count and computational complexity remained unchanged, while inference speed experienced a negligible reduction to 153 FPS. These findings confirm that the parameter-free SimAM attention mechanism enhances feature representation with virtually no additional computational burden.

When Ghost Conv was introduced independently, accuracy increased marginally by 0.6% to 88.7%. However, the parameter count decreased substantially by 30.3% to 2.65 M, computational complexity declined by 27.6% to 0.42 GFLOPs, and inference speed improved to 178 FPS. This outcome underscores the pronounced efficacy of Ghost Conv in model lightweighting, albeit with a limited impact on accuracy. The sole substitution of the Mish activation function elevated accuracy to 91.5%, a 3.4 percentage point improvement relative to the baseline. All performance metrics registered steady increments, while parameters and computational complexity remained static, and inference speed was essentially unaffected at 154 FPS. This indicates that Mish facilitates superior gradient flow and augments the model’s nonlinear expressive capacity.

Regarding module pairings, the combination of SimAM and Ghost Conv achieved an accuracy of 92.9%, exceeding Ghost Conv alone by 4.2 percentage points and SimAM alone by 0.5 percentage points. Importantly, this configuration preserved the lightweight profile—2.65 M parameters and 0.42 GFLOPs—with an inference speed of 176 FPS. This suggests that SimAM effectively compensates for the representational attenuation induced by Ghost Conv on a lightweight backbone. The pairing of Ghost Conv and Mish yielded an accuracy of 92.1%, surpassing the 88.7% of Ghost Conv alone and the 91.5% of Mish alone, yet falling slightly short of the 93.0% attained by the SimAM–Mish combination. The parameter count and computational complexity remained consistent with a lightweight architecture, with an inference speed of 174 FPS. These results imply a degree of synergy between Mish and Ghost Conv, albeit less pronounced than that observed with SimAM. The integration of SimAM and Mish produced an accuracy of 93.0% and an F1 score of 93.0%, ranking second among all pairwise combinations. However, owing to the absence of Ghost Conv, the parameter count (3.80 M) and computational complexity (0.58 GFLOPs) persisted at comparatively elevated levels.

The concurrent integration of all three modules yielded optimal performance metrics: accuracy of 94.2%, precision of 94.3%, recall of 94.2%, and an F1 score of 94.2%, corresponding to a 6.1 percentage point enhancement over the baseline. Simultaneously, the model maintained 2.65 M parameters and 0.42 GFLOPs in computational complexity—consistent with configurations dominated by Ghost Conv—while achieving a peak inference speed of 183 FPS. These findings compellingly illustrate the complementary functionality of the three modules: SimAM and Mish collectively enhance feature quality and nonlinear expressiveness, whereas Ghost Conv curtails model size and accelerates inference. Collectively, they strike an optimal trade-off between accuracy and efficiency. In conclusion, the ablation study substantiates that SimAM and Mish constitute effective strategies for elevating accuracy without incurring additional parametric or computational overhead, while Ghost Conv markedly reduces model complexity. The integrated application of these three modules secures maximal accuracy gains while preserving lightweight model characteristics, thereby corroborating the rationality and efficacy of the proposed improvement framework.

**Figure 5:**
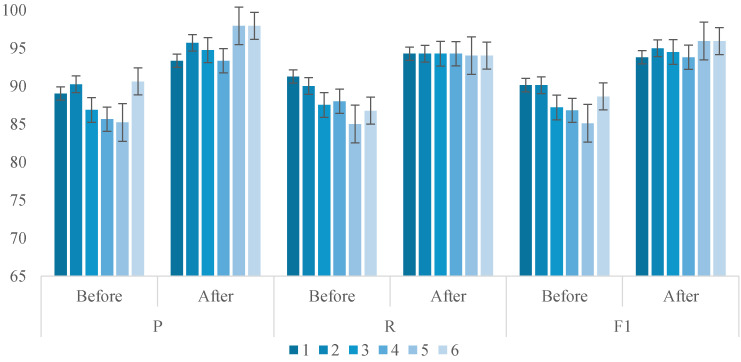
Comparison across categories before and after model improvement.

To validate the effectiveness of the improvement strategy, this study compared the precision, recall, and F1 scores across all categories before and after model optimization. As shown in **Figure 5**, the improved model achieved significant improvements in all categories, with comprehensive enhancements in all metrics, thereby validating the overall effectiveness of the improvement strategy. In terms of precision, the recognition accuracy for all categories increased substantially. Among these, the improvements for “Damaged” and ‘Intact’ were the most significant, with precision rates jumping from 85.21% and 90.60% before the improvement to 97.92%, indicating a fundamental enhancement in the model’s ability to distinguish between healthy grains and physically damaged ones. The precision rates for “Insect-damaged” and “Moldy” also increased by 5.45% and 7.87%, respectively, demonstrating a significant optimization in the model’s ability to capture subtle textural features. In terms of recall, the model’s detection capability for all defect categories has been comprehensively enhanced. Recall rates for all categories showed notable increases, particularly for lesions and sprouting, which rose by 3.00% and 6.25%, respectively, effectively reducing the false negative rate. This indicates that the improved model can more comprehensively cover real samples when handling defects with similar morphologies and blurred boundaries. In terms of the overall F1 score, the average F1 score of the improved model across the six categories rose from approximately 88.0% to 94.8%, representing a nearly 7-percentage-point leap in overall performance. Notably, the F1 scores for both “damaged” and “intact” categories exceeded 95%, reaching 95.92%, demonstrating exceptional classification balance. These results fully demonstrate that the proposed improvement method not only enhances the model’s overall accuracy but also achieves breakthrough progress in fine-grained feature differentiation and class balance, providing more reliable technical support for the automatic identification of wheat grain abnormalities.

**Figure 6:**
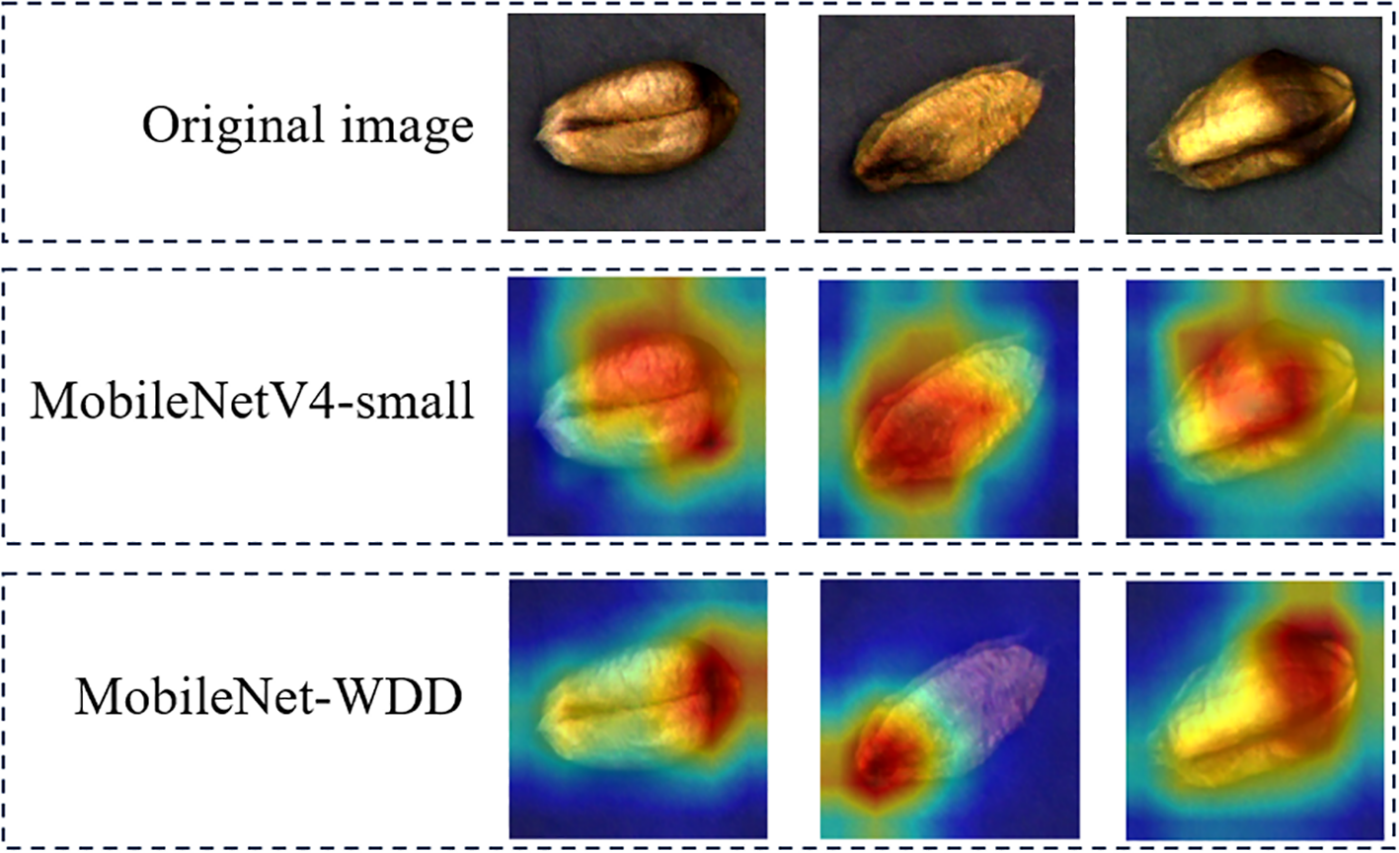
Visualization of recognition effect before and after model improvement.

Grad-CAM-based activation heatmap analysis provides an intuitive visual explanation for understanding the model’s decision-making process, making it particularly suitable for comparing differences in attention regions between the original and improved models in the task of identifying abnormal wheat. Grad-CAM calculates the gradients of the target class with respect to deep feature maps via backpropagation, computes the weights for each channel, and generates a spatial heatmap to highlight the image regions that contribute most significantly to the model’s classification decisions (Selvaraju et al., 2020). The specific results are shown in **Figure 6**. When the original MobileNetV4-small model identifies abnormal wheat images, the generated heatmap exhibits a clear lack of focus. High-response regions not only cover the areas where grain abnormalities occur but are also extensively distributed across the surfaces of normal grains and even irrelevant regions in the image background. This broad, cross-category activation indicates that the baseline model failed to effectively suppress interference from background noise and normal structures. Its learned feature representations were contaminated with redundant information weakly associated with abnormal phenotypes, resulting in unclear decision-making criteria and potentially limited generalization capabilities.

In contrast, the improved MobileNet-WDD model exhibited distinctly different attention-focused characteristics on the same test samples. In the heatmaps generated by this model, regions with high activation values are highly concentrated in a few compact and semantically distinct local areas, which precisely correspond to the exact locations where abnormal changes actually occur on the wheat grains. Meanwhile, the regions of normal grains, rachis structures, and background areas that were continuously activated in the original model have almost entirely shifted to a low-response state, with a significant increase in the proportion of cool-toned regions. This shift in attention patterns from dispersion to concentration intuitively validates the improvement strategy introduced by MobileNet-WDD. Consequently, the visualization results from Grad-CAM, from the perspective of spatial attention distribution, strongly confirm that the MobileNet-WDD model possesses the ability to precisely locate abnormal regions in the wheat anomaly detection task, thereby providing visual evidence of the model’s reliability that goes beyond single numerical metrics.

### 3.3 Comparative experiment

#### 3.3.1 Comparative experiments with other models

To further validate the overall superiority of the MobileNet-WDD model proposed in this paper on the wheat dataset, this study evaluated its performance against mainstream classification models—including MobileNetV3-S, EfficientNet-B0, GhostNet, FasterNet-T0, StarNet-S050, EfficientViM-M1, ResNet50, and DenseNet121—under identical experimental conditions. The results are shown in **Table 6**. The experimental results show that MobileNet-WDD achieved the highest accuracy of 94.2%, precision of 94.3%, recall of 94.2%, and F1 score of 94.2%, significantly outperforming all comparison models. Among lightweight models, EfficientViM-M1 achieved a precision of 87.8%, EfficientNet-B0 87.5%, StarNet-S050 87.1%, GhostNet 86.8%, FasterNet-T0 86.2%, and MobileNetV3-S 85.5%, all of which lagged significantly behind MobileNet-WDD. The classic ResNet50 and DenseNet121 achieved accuracy rates of only 86.5% and 85.9%, respectively. In terms of model efficiency, MobileNet-WDD has 2.65 million parameters, a computational complexity of 0.42 GFLOPs, and an inference speed of 183 FPS. Among the compared models, MobileNetV3-S has the smallest number of parameters at 2.54 M, but its accuracy is only 85.5%; GhostNet has 3.12 M parameters and a lower computational complexity of 0.13 GFLOPs, yet its accuracy is only 86.8%; FasterNet-T0 has 3.85 M parameters and a high computational complexity of 0.63 GFLOPs; EfficientNet-B0 has 5.28 million parameters and a computational complexity of 0.39 GFLOPs, with an inference speed of 165 FPS; whereas ResNet50 has as many as 25.56 million parameters, a computational complexity of 4.10 GFLOPs, and an inference speed of only 95 FPS. In summary, MobileNet-WDD achieves recognition accuracy far surpassing that of other comparison models while maintaining extremely low parameter counts and computational complexity, and it also offers the fastest inference speed. This fully demonstrates that the improvement strategy proposed in this paper achieves the optimal balance between lightweight design and high accuracy, enabling rapid and precise identification of wheat grains.

**Table 6:** Comparative experiments with other models.

Model	Acc (%)	P (%)	R (%)	F1 (%)	Params (M)	FLOPs	FPS
MobileNetV3-S	85.5	85.7	85.5	85.5	2.54	0.06	215
EfficientNet-B0	87.5	87.6	87.5	87.5	5.28	0.39	165
GhostNet	86.8	87.0	86.8	86.8	3.12	0.13	198
FasterNet-T0	86.2	86.4	86.2	86.2	3.85	0.63	172
StarNet-S050	87.1	87.3	87.1	87.1	3.40	0.55	158
EfficientViM-M1	87.8	87.9	87.6	87.8	4.10	0.68	145
ResNet50	86.5	86.5	86.5	86.4	25.56	4.10	95
DenseNet121	85.9	86.4	85.9	86.0	7.98	2.88	110
MobileNet-WDD	94.2	94.3	94.2	94.2	2.65	0.42	183

**Figure 7:**
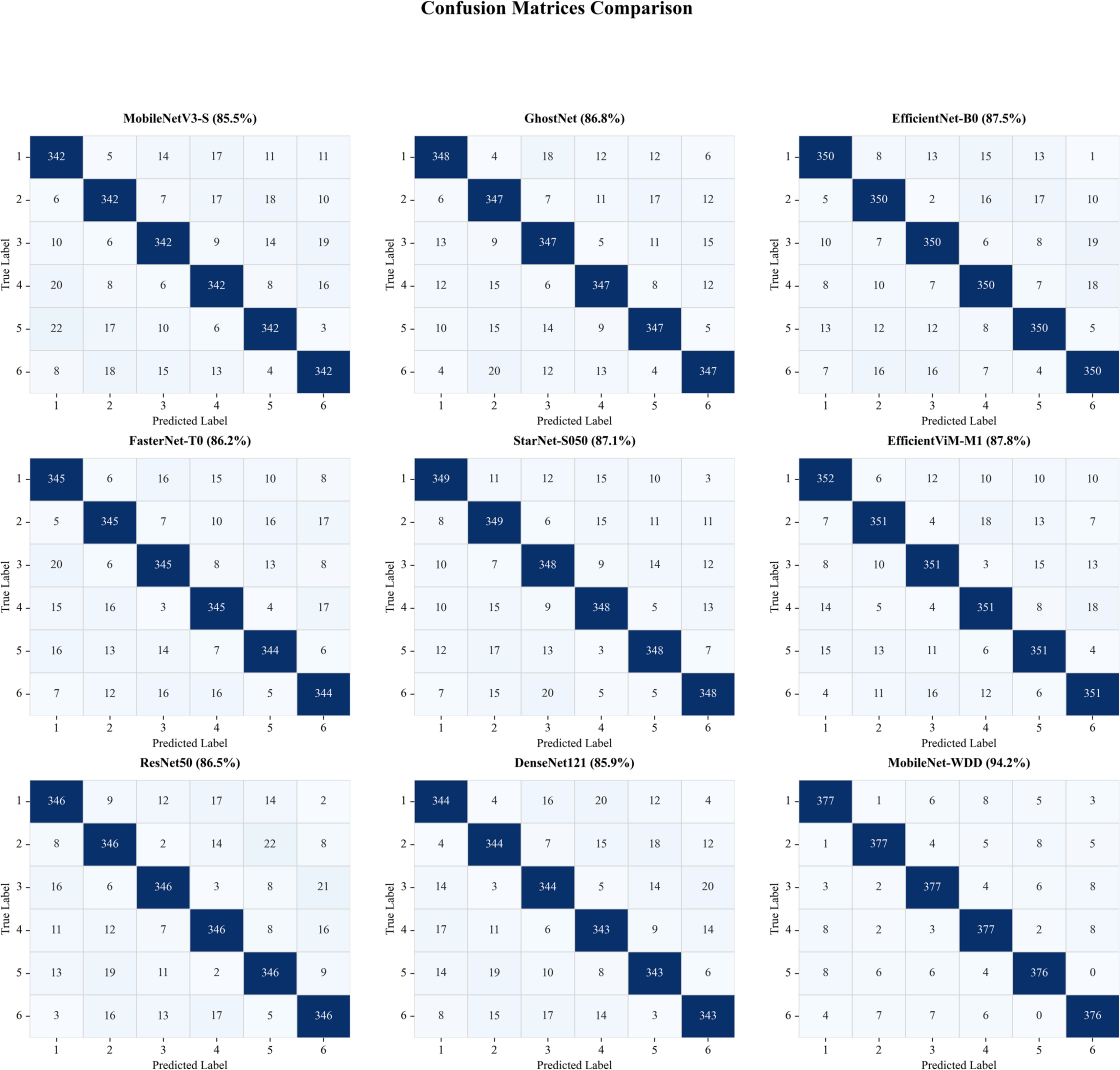
Confusion matrices for the nine models in the comparative experiment.

This study evaluated the performance of deep learning models across six categories of wheat grains. By comparing the confusion matrices of nine mainstream network architectures, the results showed that the MobileNet-WDD model performed best with an accuracy of 94.2%, significantly outperforming the other models. The specific results are shown in **Figure 7**. This model demonstrates balanced classification accuracy across all categories. Notably, when distinguishing between intact and damaged grains, its accuracy approaches 98%, indicating exceptional feature extraction capabilities and robustness, enabling effective differentiation between morphologically similar healthy and damaged samples.

In contrast, classic models such as MobileNetV3-S, ResNet50, and DenseNet121 all had accuracy rates below 86.5%. They exhibited significant confusion in distinguishing fine-grained features—such as lesions versus sprouting and insect damage versus breakage—and were unable to meet the requirements for high-precision detection. GhostNet and EfficientNet-B0 performed averagely, with misclassifications primarily concentrated between the mold and sprouting categories, indicating limitations in handling defects with similar color and texture. Although FasterNet-T0 and StarNet-S050 showed slight improvements, they still failed to fundamentally resolve the issue of feature overlap between categories. Although EfficientViT-M1 achieves high precision in some categories, its recall rate is relatively low, posing a risk of missed detections.

In summary, MobileNet-WDD not only establishes a leading advantage in overall recognition accuracy but also balances recall with high precision, validating its effectiveness and practical value in the task of automated detection of wheat grain appearance quality. This provides reliable algorithmic support for future intelligent agricultural sorting.

**Figure 8:**
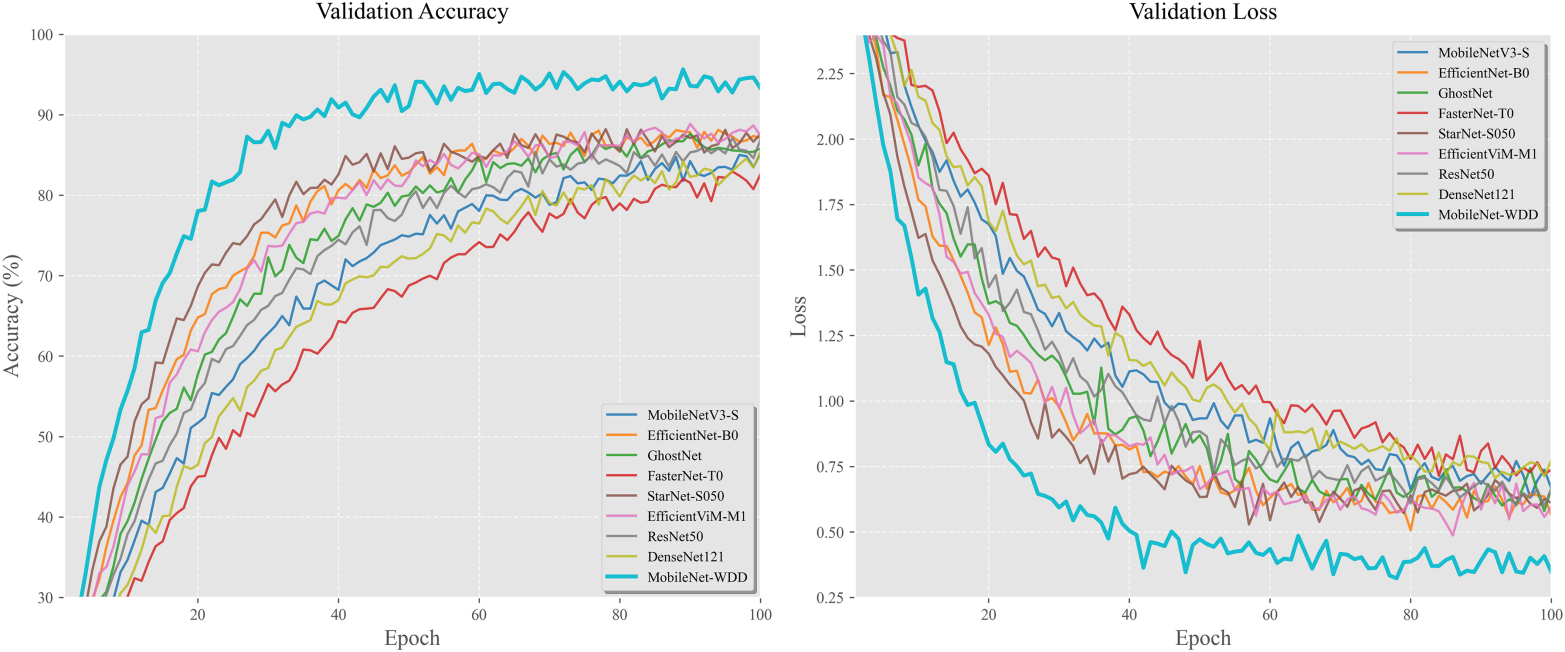
Accuracy and loss curves for the nine models in the comparative experiment on the validation set.

As shown in **Figure 8**, the validation set accuracy and loss curves for nine models in the wheat grain anomaly detection task clearly reveal significant differences among various network architectures in terms of convergence speed, stability, and final performance. MobileNet-WDD demonstrates a clear advantage in both curves. Its validation accuracy curve rises most rapidly, quickly surpassing 80% in the early stages of training and reaching a convergence plateau around the 40th epoch, ultimately stabilizing at a high level above 94%. At the same time, its validation loss curve exhibits the steepest descent and the lowest values, stabilizing within the range of 0.25 to 0.50. This indicates that the model not only learns rapidly but also maintains exceptional stability during training, enabling it to efficiently capture data features and achieve optimal generalization capabilities.

In contrast, the other comparison models exhibited varying degrees of limitations. The accuracy curves of MobileNetV3-S, ResNet50, and DenseNet121 were primarily distributed in the low-to-mid range of 80% to 85%, and their loss curves declined slowly with significant fluctuations. This suggests that these models face difficulties in extracting subtle defect features from wheat grains and exhibit lower convergence efficiency. Although medium-sized models such as GhostNet and EfficientNet-B0 achieved higher final accuracy than the aforementioned models, they exhibited significant fluctuations during training. In particular, between the 60th and 100th training epochs, the accuracy curves fluctuated frequently, reflecting an optimization process that was not sufficiently smooth.

It is worth noting that while some models, such as EfficientViT-M1, achieved acceptable final accuracy, they exhibited intensified accuracy fluctuations during the late training stages, suggesting a potential risk of overfitting or getting trapped in a local optimum. Overall, MobileNet-WDD, with its unique architectural design, achieves the lowest validation loss and highest classification accuracy while ensuring extremely fast convergence. This fully demonstrates its superiority and stability in handling fine-grained agricultural image classification tasks, making it the optimal choice that balances efficiency and performance.

#### 3.3.2 Comparative experiment of different attention mechanisms

To validate the advantages of the SimAM attention mechanism over other mainstream attention mechanisms, this study used MobileNetV4 small as the baseline network and conducted comparative experiments by integrating SE, CBAM, ECA, LSKA, EMA, and SimAM modules, respectively. The results are shown in **Table 7**. The experiments demonstrate that SimAM achieves the optimal balance between accuracy and efficiency, with an accuracy, precision, recall, and F1 score of 92.4%, 92.2%, 92.6%, and 92.4%, respectively. It has 3.80 million parameters, a computational complexity of 0.58 GFLOPs, and an inference speed of 153 FPS. LSKA achieved a slightly higher accuracy of 92.6%, but its number of parameters increased to 3.82 M, computational complexity rose to 0.61 GFLOPs, and inference speed dropped to 148 FPS, making it significantly less efficient than SimAM. EMA achieved an accuracy of 92.1%, with 3.84 million parameters, a computational complexity of 0.60 GFLOPs, and an inference speed of 149 FPS; all efficiency metrics were inferior to those of SimAM. CBAM achieved an accuracy of 91.6%, but with a high parameter count of 3.88 million, a computational complexity of 0.62 GFLOPs, and an inference speed of only 145 FPS, making it the method with the largest parameter count and slowest speed. SE and ECA achieved accuracy rates of 91.2% and 90.8%, respectively. Although ECA shares the same number of parameters and computational complexity as SimAM and has a similar inference speed, its accuracy is 1.6 percentage points lower. In summary, when based on MobileNetV4 small, SimAM achieves near-optimal accuracy while maintaining the lowest number of parameters and computational complexity, and it also offers the fastest inference speed. This is attributed to its mechanism for generating 3D attention weights without requiring additional parameters, thereby avoiding the parameter bloat and computational latency associated with traditional attention modules.

**Table 7:** Comparison of different attention mechanisms.

Attention mechanism	Acc (%)	P (%)	R (%)	F1 (%)	Params (M)	FLOPs	FPS
SE	91.2	91.0	91.4	91.2	3.85	0.59	150
CBAM	91.6	91.4	91.8	91.6	3.88	0.62	145
ECA	90.8	90.6	91.0	90.8	3.80	0.58	152
LSKA	92.6	92.4	92.8	92.6	3.82	0.61	148
EMA	92.1	91.9	92.3	92.1	3.84	0.60	149
SimAM	92.4	92.2	92.6	92.4	3.80	0.58	153

#### 3.3.3 Comparison of different activation functions

**Figure 9:**
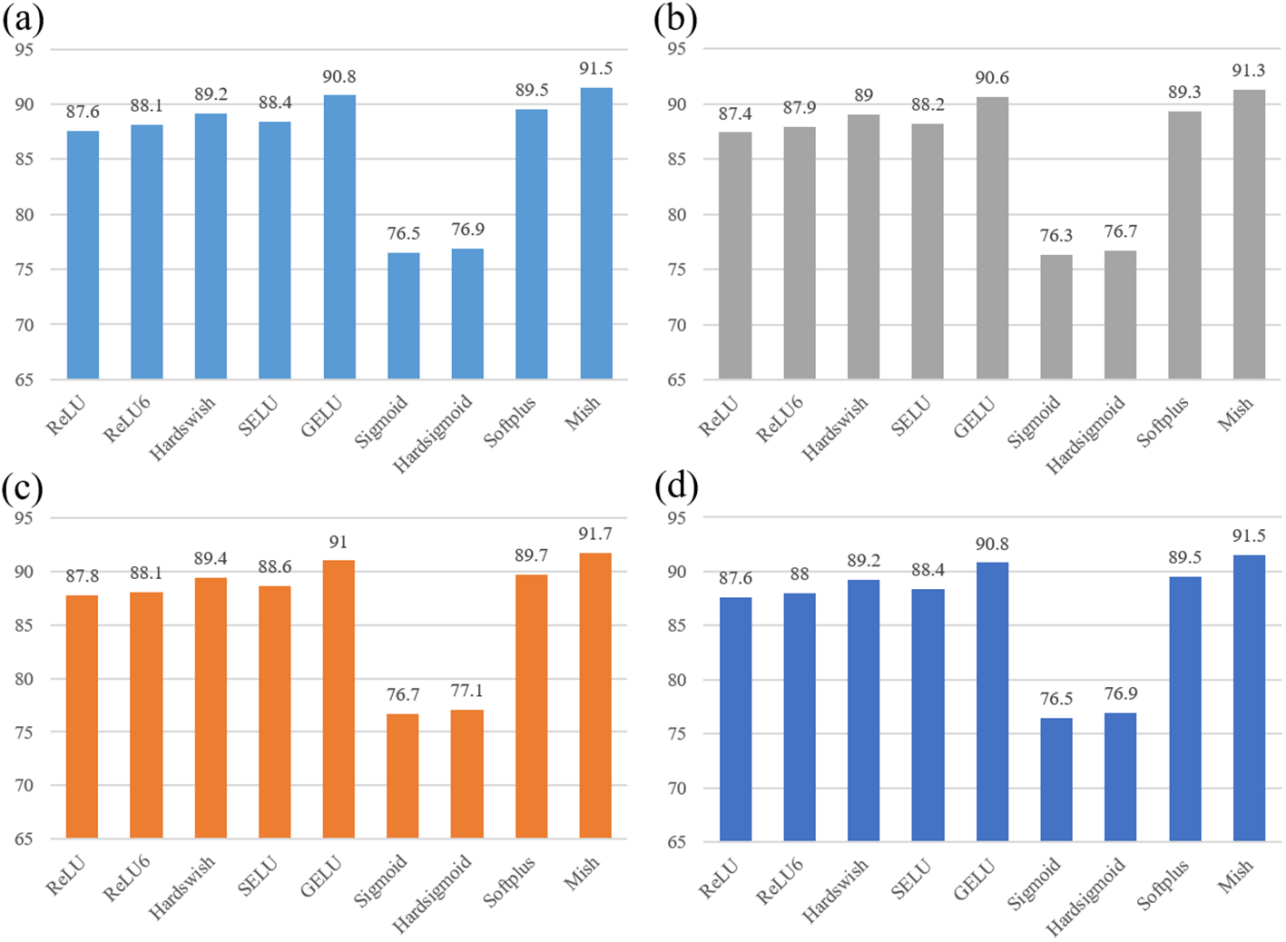
Comparison of different activation functions: **(a)** Accuracy Comparison, **(b)** Precision comparison, **(c)** Recall comparison, **(d)** F1 score comparison.

To validate the effectiveness of the Mish activation function within the MobileNetV4-small framework, this study compared it with commonly used activation functions such as ReLU, ReLU6, Hardswish, SELU, GELU, Sigmoid, Hardsigmoid, and Softplus. The results are shown in **Figure 9**. The experimental results show that Mish achieved the best performance, with accuracy, precision, recall, and F1 score reaching 91.5%, 91.3%, 91.7%, and 91.5%, respectively. The corresponding metrics for traditional ReLU were 87.6%, 87.4%, 87.8%, and 87.6%, indicating that Mish improved by approximately 3.9 percentage points across all metrics. GELU achieved a precision of 90.8%, which is still approximately 0.7 percentage points lower than Mish; Hardswish and Softplus had precisions of 89.2% and 89.5%, respectively, also falling short of Mish. Sigmoid and Hardsigmoid performed the worst, with precisions of only 76.5% and 76.9%, indicating that saturating activation functions are not suitable for this task. These results clearly demonstrate that, when using MobileNetV4 small as the base network, the Mish activation function—with its smooth, non-monotonic characteristics—can more effectively facilitate gradient flow and preserve negative information, thereby enhancing the model’s nonlinear fitting capability and overall recognition accuracy.

## 4 Discussion

This study addresses the challenge of automatically classifying six types of wheat grain appearance conditions by developing an improved MobileNet-WDD deep learning model. Through a systematic analysis of the confusion matrix, training convergence curves, and performance metrics before and after the improvement, the results demonstrate that the model exhibits significant advantages in terms of recognition accuracy, training efficiency, and robustness. The following sections will provide an in-depth discussion from the perspectives of feature extraction mechanisms, training dynamics, validation of the improvement strategy’s effectiveness, and agricultural engineering application value.

From the perspective of feature extraction and classification mechanisms, the detection of appearance defects in wheat grains is a typical fine-grained image classification task. The core challenge lies in the high inter-class similarity among different defect categories. For example, both disease spots and sprouting manifest as localized color changes, while insect damage and breakage involve contour loss and fractures in their morphology. Traditional convolutional neural networks, such as ResNet50 and DenseNet121, often struggle to capture these subtle textural differences due to the limitations of their fixed convolutional kernel receptive fields, leading to significant cross-class misclassifications in the confusion matrix. In contrast, MobileNet-WDD significantly enhances sensitivity to local fine-scale features by introducing targeted structural improvements. Experimental data show that the model achieves a precision rate of nearly 98% for both damaged and intact seeds, indicating that it has successfully constructed a feature space with high discriminative power for the boundary between “healthy” and “damaged” states. This high-precision discrimination capability is attributed to the model’s enhanced extraction of edge gradients and textural details in its deep layers, enabling it to effectively filter out interference from natural gloss variations on the grain surface and precisely identify key features indicative of pathological or physical damage.

The accuracy and loss curves during training reveal the model’s excellent convergence speed and stability. In experiments comparing nine mainstream network architectures, MobileNet-WDD demonstrated the notable characteristics of “fast convergence and low loss.” Its validation accuracy curve shows a steep upward trend from the early stages of training, surpassing the 90% threshold and entering a stable plateau after only about 40 training epochs, whereas models such as EfficientNet-B0 require longer training periods and exhibit greater fluctuations. Meanwhile, its loss function value rapidly decreases and stabilizes in the low range of 0.25 to 0.50, without showing any significant overfitting fluctuations. These results indicate that the improved model architecture, while maintaining feature extraction capabilities, significantly optimizes the smoothness of the parameter search space, making the gradient descent process more efficient. This rapid convergence is crucial for model iteration in agricultural scenarios, meaning that in practical deployment, the model can complete training and updates with lower computational costs and in less time, adapting to the detection needs of different wheat varieties or batches.

Further analysis of the changes in various metrics before and after the model improvement allows for a quantitative assessment of the specific contributions of the optimization strategy. Comparative data show that the improved model achieved a comprehensive leap in precision, recall, and F1 scores across all six categories. In particular, for the “damaged” and “intact” categories, precision increased by 12.71% and 7.32%, respectively, while recall improved by 9.00% and 7.25%, respectively, with F1 scores exceeding 95% in both cases. This significant performance leap demonstrates that the improvements effectively resolved the original model’s issues of false negatives and false positives when processing samples with complex morphologies. Furthermore, for the more challenging categories of insect damage and mold, the F1 scores increased by 4.85% and 7.31%, respectively, proving that while the model enhanced its ability to identify significant defects, it did not sacrifice sensitivity to minor blemishes, thereby achieving a balanced improvement in overall performance.

From the perspective of agricultural engineering applications, the high performance of MobileNet-WDD holds profound practical significance. In modern grain processing and storage operations, high-speed, non-destructive quality sorting is critical to ensuring food security. Traditional machine vision methods are often limited by algorithmic robustness, making it difficult to meet the real-time detection demands of high-speed conveyor belts. The model proposed in this study not only meets stringent sorting standards in terms of accuracy, but its lightweight design also enables deployment on edge computing devices. High recall ensures that substandard grains are intercepted to the greatest extent possible, guaranteeing product quality upon shipment; high precision reduces false rejection rates, minimizes grain loss, and directly enhances economic benefits. In summary, by addressing key technical bottlenecks such as the difficulty of fine-grained feature extraction and slow convergence, the MobileNet-WDD model provides an efficient and reliable intelligent solution for wheat quality inspection, offering broad prospects for widespread application.

## 5 Conclusion

In response to the need for automated inspection of wheat grain appearance quality, this study proposes an improved MobileNet-WDD deep learning model. Systematic experimental validation demonstrates that the model achieves outstanding recognition performance while maintaining extremely low resource consumption. Specifically, the model achieved a high accuracy rate of 94.2% on the test set, accurately distinguishing wheat grains with lesions, insect damage, mold, sprouting, damage, and intact conditions, thereby meeting the high-precision requirements of automated agricultural sorting. Notably, compared to traditional mainstream models, MobileNet-WDD significantly reduces both the number of parameters and computational load, demonstrating exceptional computational efficiency and lightweight advantages. This greatly facilitates the model’s deployment on resource-constrained edge computing devices or embedded systems, effectively lowering hardware costs and power consumption. This study confirms that MobileNet-WDD enhances detection accuracy while maintaining model compactness and practicality, offering a highly competitive solution for the efficient, non-destructive inspection of wheat quality. Future work will focus on further optimizing the model’s generalization capabilities in complex backgrounds and promoting its integration into actual production lines.

## Data Availability

The original contributions presented in the study are included in the article/supplementary material. Further inquiries can be directed to the corresponding authors.
